# *Notes from the Field:* The National Wastewater Surveillance System’s Centers of Excellence Contributions to Public Health Action During the Respiratory Virus Season — Four U.S. Jurisdictions, 2022–23

**DOI:** 10.15585/mmwr.mm7248a4

**Published:** 2023-12-01

**Authors:** Diana Valencia, Alexander T. Yu, Allison Wheeler, Loren Hopkins, Ian Pray, Libby Horter, Duc J. Vugia, Shannon Matzinger, Lauren Stadler, Nathan Kloczko, Michael Welton, Stephanie Bertsch-Merbach, Kaavya Domakonda, Dagmara Antkiewicz, Hannah Turner, Chad Crain, Anthony Mulenga, Martin Shafer, Judith Owiti, Rebecca Schneider, Kayley H. Janssen, Marlene K. Wolfe, Sandra L. McLellan, Alexandria B. Boehm, Adélaïde Roguet, Bradley White, Melissa K. Schussman, Madhura S. Rane, Jocelyn Hemming, Caroline Collins, Andrew Abram, Elisabeth Burnor, Ryan Westergaard, Jessica N. Ricaldi, John Person, Nicole Fehrenbach

**Affiliations:** ^1^Division of Infectious Disease Readiness & Innovation, National Center for Emerging and Zoonotic Infectious Diseases, CDC; ^2^California Department of Public Health; ^3^Colorado Department of Public Health & Environment; ^4^Houston Health Department, Houston, Texas; ^5^Rice University, Houston, Texas; ^6^Wisconsin Department of Health Services; ^7^Career Epidemiology Field Officer Training Program, CDC; ^8^Wisconsin State Laboratory of Hygiene; ^9^University of Wisconsin-Madison, Madison, Wisconsin; ^10^Gangarosa Department of Environmental Health, Rollins School of Public Health, Emory University, Atlanta, Georgia; ^11^School of Freshwater Sciences, University of Wisconsin-Milwaukee, Milwaukee, Wisconsin; ^12^Department of Civil & Environmental Engineering, School of Engineering and Doerr School of Sustainability, Stanford University, Stanford, California; ^13^Verily Life Sciences, San Francisco, California.

Wastewater surveillance (WWS), the systematic detection of infectious agents in wastewater, provided a valuable tool for monitoring SARS-CoV-2 circulation during the COVID-19 pandemic; surveillance has expanded from 20 to 53 jurisdictions across the United States, with increasing capacity to test for more respiratory pathogens (*1*,*2*). This report highlights the use of wastewater data by the four National Wastewater Surveillance System’s (NWSS) Centers of Excellence (California; Colorado; Houston, Texas; and Wisconsin) to guide public health action during the 2022–23 respiratory disease season. This activity was reviewed by CDC, deemed not research, and was conducted consistent with applicable federal law and CDC policy.*

## Implementation and Action

Four CDC-funded NWSS Centers of Excellence were established during 2021–22. During 2022–23, wastewater sampling covered a large proportion of the sites’ populations: 94% (Houston, Texas), 67% (California), 65% (Colorado), and 50% (Wisconsin). Implementation and data usage varied by locality (Box).

BOX **Examples of implemented public health actions related to respiratory viruses — National Wastewater Surveillance System’s Centers of Excellence, four U.S. jurisdictions, 2022–23**

**Local level**

**Colorado (Denver metro)**

- Biweekly sampling for EV-D68 in the Denver metro area to guide and collaborate with health system and pediatric providers during 2022–23 respiratory season
- Biweekly sampling of three sewersheds in Denver, covering 1.2 million residents
- Three statewide alerts to guide hospitals and providers about increases in cases to plan surge staffing and resource allocation as a result of the EV-D68 syndromic surveillance alarm
- Wastewater testing performed retrospectively and showed an increasing trend 1 month before the syndromic surveillance alarm; wastewater testing is now part of the EV-D68 multimodal surveillance model

**Houston**

- Weekly wastewater monitoring for SARS-CoV-2, influenza, and RSV in K–12 schools to detect outbreaks during 2022–23 school year
- Weekly sampling of 122 sampling sites (39 wastewater treatment plants, 14 lift stations, and 69 manholes) covering 2.17 million residents
- Forty-eight manholes sampled from 48 schools serving approximately 34,000 students (1.6% of total National Wastewater Surveillance System sewershed and 18.6% of the city’s school population)
- Data shared on public dashboard (>362,000 views) and 27 reports to schools
- Data provided for vaccine clinic deployment that administered 1,058 COVID-19 and influenza vaccine doses to 992 students as of May 2023

**State level**

**California**

- Daily to weekly sampling of 98 sewersheds to estimate SARS-CoV-2 variants, RSV, and influenza disease activity in 41 counties (67% of residents [26 million persons] within sampled sewersheds) during 2022–23 respiratory disease season
- Messaging to local public health and the public, including through weekly summary reports and dashboards
- Local public health messaging to health care providers and the community for awareness and to support recommendations (e.g., vaccines and masking) including through media, press reports, and dashboards

**Wisconsin**

- Weekly or biweekly monitoring for SARS-CoV-2 and variants at wastewater treatment facilities
  - Forty-three sampling sites covering 2.93 million residents
  - Data shared on public dashboards with alert notification (>250,000 views), genomic sequencing dashboard (>6,000 views), and weekly stakeholder reports
- Weekly or biweekly monitoring for influenza A and B and RSV at wastewater treatment facilities
  - Twenty sampling sites covering 2.48 million residents
  - Internal monitoring dashboard; public dashboard and weekly stakeholder reports in development

**Abbreviations:** EV = enterovirus; RSV = respiratory syncytial virus.

**Public Health Actions at the Local Level**

**Colorado.** To help guide local public health action in Colorado, three Denver sewersheds, covering 1.2 million residents, submitted biweekly wastewater samples. Retrospective analyses indicated that WWS detected enterovirus D68 (EV-D68) ≤1 month before syndromic and clinical laboratory signals. This finding led public health officials in Colorado to implement wastewater testing for EV-D68 as part of the enterovirus surveillance model to provide an early warning system for health care surge planning during respiratory virus season.

**Houston.** This metropolitan area WWS included 122 sampling sites covering 2.17 million residents. Sampling and testing for SARS-CoV-2, influenza virus, and respiratory syncytial virus (RSV) from 48 manholes associated with selected schools provided data to support strategically deployed school vaccination clinics (1,058 COVID-19 and influenza vaccine doses administered), empowered staff members in 48 schools to implement respiratory disease prevention strategies through school reports, and increased public awareness through a dashboard, which recorded approximately 350,000 views as of 2023. Recently, alert notifications were launched (698 registered users are associated with 46 schools) to inform users about identification of surges in respiratory viruses.^†^

**Public Health Actions at the State Level**

**California.** In California, WWS supports local health department decision-making, and has been used to provide tailored metrics and messaging to communities, providers, and health care systems to improve awareness and preparedness. Activities included daily to weekly sampling of 98 sewersheds to detect SARS-CoV-2 variants, RSV, and influenza virus in 41 counties, covering approximately 26 million residents; results are communicated via dashboards and weekly reports.

**Wisconsin.** Wisconsin performed daily to weekly sampling for SARS-CoV-2 at 43 sampling sites covering approximately 2.93 million residents. As of 2023, WWS data were shared on a public dashboard with alert notifications (>250,000 views), a genomic sequencing dashboard (>6,000 views), and weekly reports to local health departments and water treatment utility companies. Wisconsin’s genomic sequencing dashboard has become an important tool for identifying and monitoring SARS-CoV-2 variants in wastewater, in some cases identifying variants (e.g., BA.5 and XBB) before detection through clinical surveillance (e.g., case reports and hospitalizations). Twenty sites covering approximately 2.48 million residents, were sampled for influenza viruses and RSV, and data were monitored on an internal dashboard. Wastewater concentrations for these viruses were highly correlated with emergency department visits in Wisconsin during 2022–23 (*3*), forming the basis for continued monitoring through the 2023–24 respiratory disease season.

## Preliminary Conclusions and Actions

NWSS Centers of Excellence have reported correlation between WWS data and clinical surveillance with WWS allowing localized, timely coverage, and in some situations, valuable lead time notification. In Wisconsin, WWS detected increases in influenza and RSV weeks before increases in related emergency department visits were observed (*3*–*5*). NWSS data, together with clinical surveillance data, have guided jurisdictional partner decisions regarding allocation of resources, deployment of vaccination clinics, updating clinical guidance, and sending respiratory disease notifications and alerts when trends exceed baseline thresholds. NWSS Centers of Excellence have developed public-facing and internal pathogen data dashboards that provide metrics for public health partners and the communities they serve. During the 2022–23 respiratory disease season, NWSS Centers of Excellence translated WWS data into real-time public health action for multiple respiratory pathogens, highlighting the contribution of WWS in monitoring disease circulation and helping guide public health response.

## Footnotes

* 45 C.F.R. part 46.102(l)(2), 21 C.F.R. part 56; 42 U.S.C. Sect. 241(d); 5 U.S.C. Sect. 552a; 44 U.S.C. Sect. 3501 et seq.

† Centers of Excellence dashboard links. Houston (https://covidwwtp.spatialstudieslab.org, https://www.houstonhealth.org/services/wastewater), Colorado (https://cdphe.colorado.gov/covid-19/wastewater-trends, https://cdphe.maps.arcgis.com/apps/dashboards/d79cf93c3938470ca4bcc4823328946b), California (https://www.cdph.ca.gov/Programs/CID/DCDC/Pages/COVID-19/CalSuWers-Dashboard.aspx), and Wisconsin (https://www.dhs.wisconsin.gov/covid-19/wastewater.htm, https://dataportal.slh.wisc.edu/sc2-ww-dashboard).

## References

[1] Kirby AE, Walters MS, Jennings WC, Using wastewater surveillance data to support the COVID-19 response—United States, 2020–2021. MMWR Morb Mortal Wkly Rep 2021;70:1242–4. 10.15585/mmwr.mm7036a2 34499630 PMC8437053

[2] Boehm AB, Wolfe MK, White BJ, Hughes B, Duong D, Bidwell A. More than a tripledemic: influenza A virus, respiratory syncytial virus, SARS-CoV-2, and human metapneumovirus in wastewater during winter 2022–2023. Environ Sci Technol Lett 2023;10:622–7. 10.1021/acs.estlett.3c00385 37577361 PMC10413932

[3] DeJonge PM, Adams C, Pray I, Wastewater surveillance data as a complement to emergency department visit data for tracking incidence of influenza A and respiratory syncytial virus—Wisconsin, August 2022–March 2023. MMWR Morb Mortal Wkly Rep 2023;72:1005–9. 10.15585/mmwr.mm7237a2 37708080 PMC10511267

[4] Li X, Liu H, Gao L, Wastewater-based epidemiology predicts COVID-19-induced weekly new hospital admissions in over 150 USA counties. Nat Commun 2023;14:4548. 10.1038/s41467-023-40305-x 37507407 PMC10382499

[5] Varkila MRJ, Montez-Rath ME, Salomon JA, Use of wastewater metrics to track COVID-19 in the US. JAMA Netw Open 2023;6:e2325591. 10.1001/jamanetworkopen.2023.25591 37494040 PMC10372707

